# Supplementary material for: Dimensionality reduction by UMAP to visualize physical and genetic interactions
Source: Nat Commun. 2020 Mar 24;11:1537. doi: 10.1038/s41467-020-15351-4 (PMC7093466; doi:10.1038/s41467-020-15351-4)
Supplement: Supplementary file 1 — Supplementary Information [file 41467_2020_15351_MOESM1_ESM.pdf]

Supplementary Information

Dimensionality reduction by UMAP to visualize physical and genetic interactions

Dorrity et al.

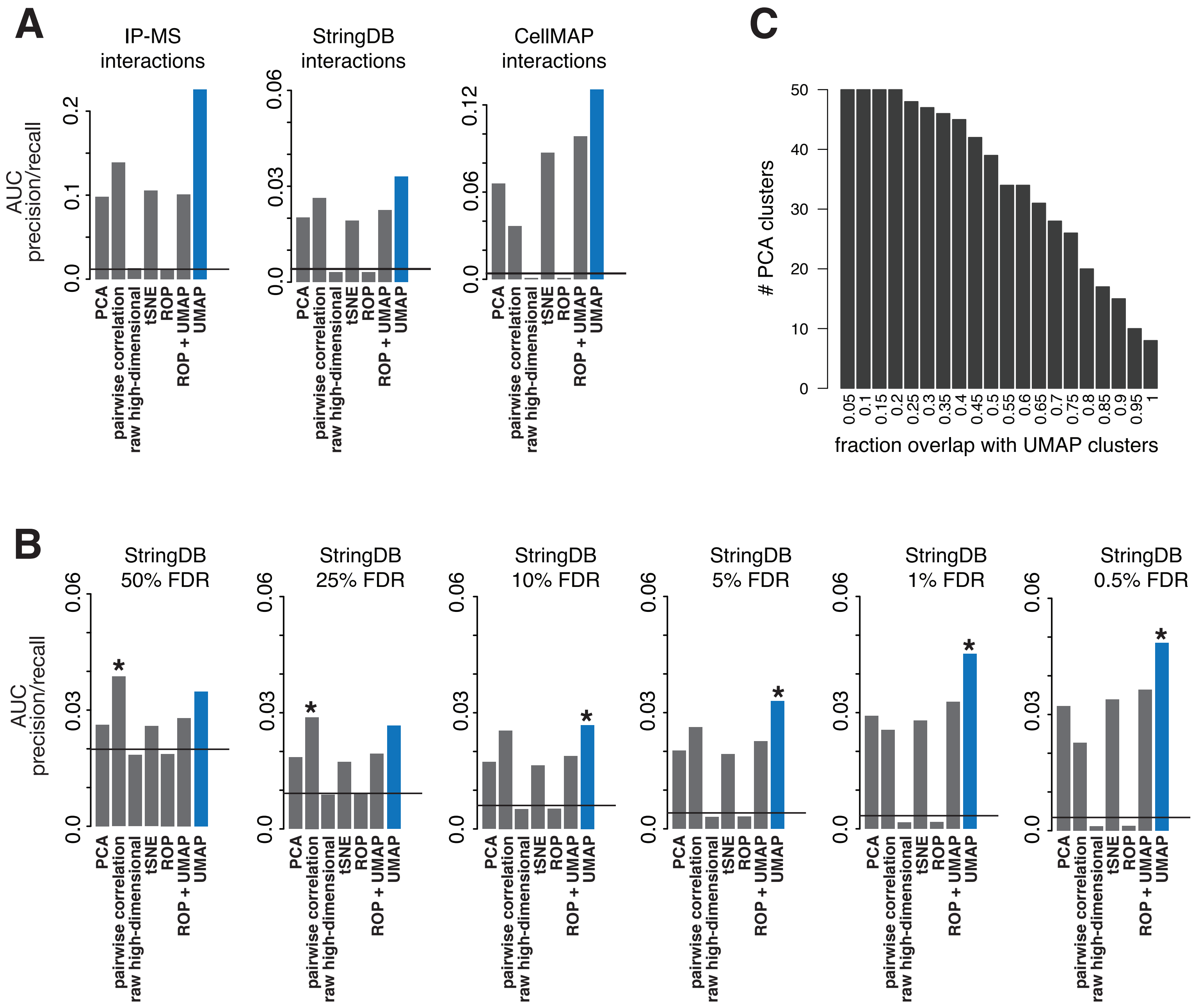

Supplementary Figure 1. UMAP adds value in identification of true interactions compared to other methods. (A) Values for area under the curves (AUC) for precision/recall curves used to benchmark distance metrics for three gold-standard interaction datasets. UMAP substantially outperforms other metrics in the identification of true protein-protein interactions. Black lines show expectations of a random test. (C) Benchmarking of distance metrics over several confidence cutoffs for true interactions as defined by StringDB. In higher FDR sets, pairwise correlations outperforms our UMAP method, but not in the higher confidence interaction sets. Black lines show expectations of a random test, and asterisks (*) define the top performer in each set. (B) Clustering to the same total number of clusters in PCA space returns few clusters strongly overlapping the clusters in UMAP space.

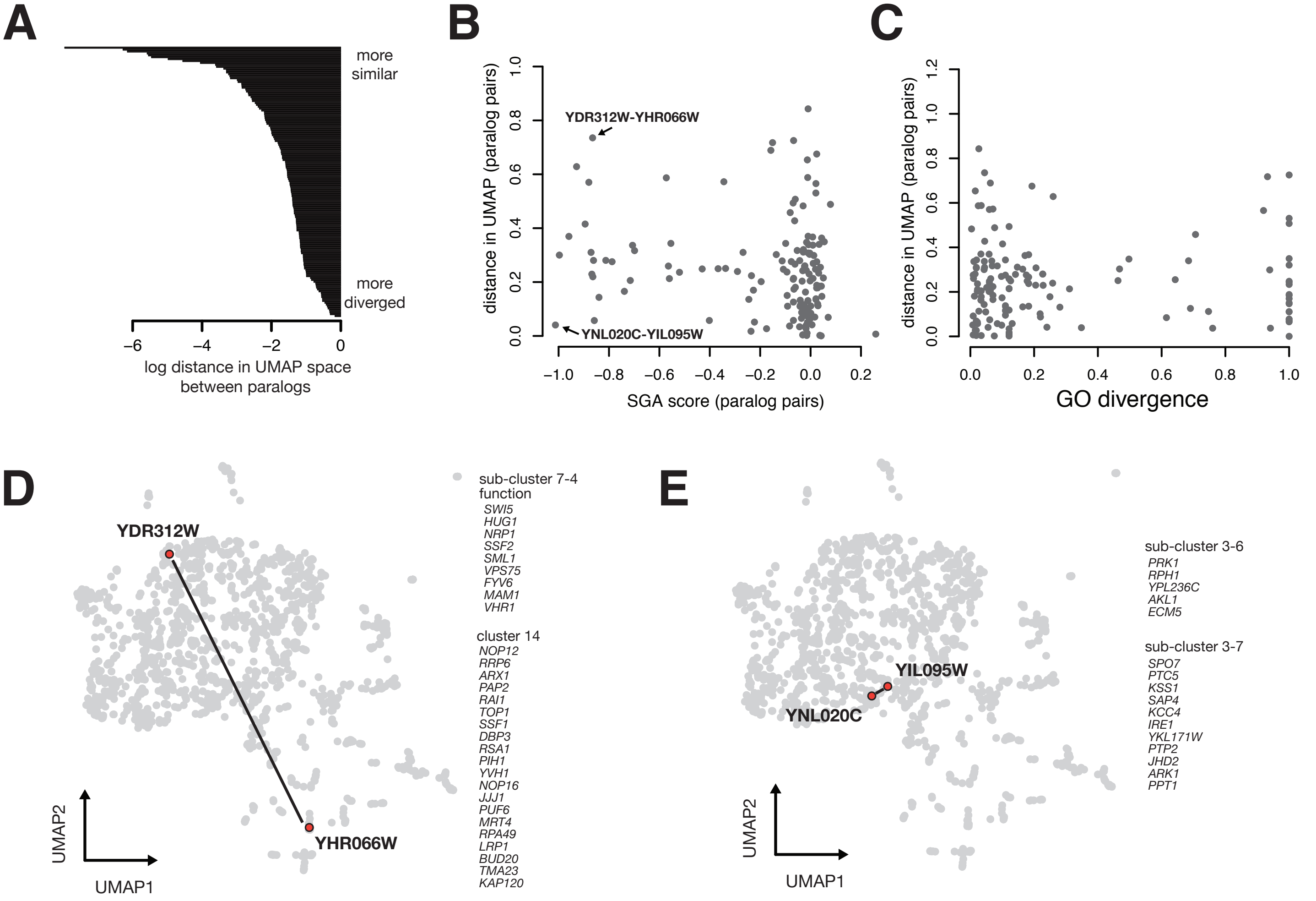

Supplementary Figure 2. Convergent and divergent function of paralogous gene pairs defined by UMAP distance. (A) Barplot showing log distance in UMAP space between 151 pairs of paralagous gene deletions. (B) Each paralog pair's UMAP distance plotted against the experimentally-determined synthetic genetic interaction score (briefly, a more negative score on the SGA axis indicates that the double mutant showed a larger cellular fitness defect than the combined additive effect of each single mutants). Two paralog pairs are indicated, and their distance in UMAP space is displayed in (D) and (E). (C) Each paralog pair's UMAP distance plotted against a metric for paralog divergence calculated using similarlity of GO term annotation. While a low score in the GO divergence metric suggests that paralog pairs have less diverged functions, many of these pairs are far from each other in UMAP space, suggesting that these paralogs show more divergent function than predicted by the GO metric. (D) Full 1484 gene deletion UMAP as in Figure 1A, with a divergent paralog pair (*SSF1* and *SSF2*) highlighted. Genes contained in the same cluster as each paralog are listed; the *SSF1* cluster (7-4) contains many genes required for ribosome biogenesis, while the *SSF2* cluster (14) contains genes involved in DNA damage. (E) Full UMAP with a convergent paralog pair (*ARK1* and *PRK1*) highlighted. Genes contained in the same cluster as each paralog are listed.
