# Supplementary material for: Dimensionality reduction by UMAP to visualize physical and genetic interactions
Source: Nat Commun. 2020 Mar 24;11:1537. doi: 10.1038/s41467-020-15351-4 (PMC7093466; doi:10.1038/s41467-020-15351-4)
Supplement: Supplementary file 2 — Description of Additional Supplementary Files [file 41467_2020_15351_MOESM2_ESM.pdf]

Description of Additional Supplementary Files

File Name: Supplementary Data 1
Description: Differentially-expressed genes in each UMAP cluster. For each of the 171 clusters (indicated in “Cluster” column), the following table lists the 250 most differentially-expressed genes (see Methods) ordered by p-value adjusted for multiple comparisons with the Benjamini-Hochberg procedure. For each differentially expressed gene, the magnitude of the expression increase within the cluster (“clust.mean.expr”) is given along with the background expression across all clusters (“bg.mean.expr”).
